# Evaluation of Intussusception After Oral Monovalent Rotavirus Vaccination in South Africa

**DOI:** 10.1093/cid/ciz431

**Published:** 2019-05-24

**Authors:** Michelle J Groome, Jacqueline E Tate, Marion Arnold, Milind Chitnis, Sharon Cox, Corné de Vos, Mari Kirsten, Susanna M le Grange, Jerome Loveland, Sello Machaea, Ashwini Maharaj, Nick Andrews, Shabir A Madhi, Umesh D Parashar

**Affiliations:** 1 Medical Research Council, Respiratory and Meningeal Pathogens Research Unit, Faculty of Health Sciences; 2 Department of Science and Technology/National Research Foundation, Vaccine Preventable Diseases, University of the Witwatersrand, Johannesburg, South Africa; 3 Centers for Disease Control and Prevention, Atlanta, Georgia; 4 Red Cross War Memorial Children’s Hospital, University of Cape Town; 5 Tygerberg Hospital, University of Stellenbosch, Cape Town; 6 East London Hospital Complex, Walter Sisulu University; 7 Steve Biko Academic Hospital/Kalafong Hospital, University of Pretoria; 8 Universitas Hospital, University of the Free State, Bloemfontein; 9 Chris Hani Baragwanath Academic Hospital/Charlotte Maxeke Johannesburg Academic Hospital, University of the Witwatersrand, Johannesburg; 10 Inkosi Albert Luthuli Hospital, University of Kwa-Zulu Natal, Durban, South Africa; 11 Statistics, Modelling and Economics Department, Public Health England, London, United Kingdom

**Keywords:** intussusception, rotavirus vaccine, infant, safety

## Abstract

**Background:**

Postlicensure studies have shown an association between rotavirus vaccination and intussusception. We assessed the risk of intussusception associated with Rotarix (RV1) administration, at 6 and 14 weeks of age, in an upper-middle-income country, South Africa.

**Methods:**

Active prospective surveillance for intussusception was conducted in 8 hospitals from September 2013 through December 2017. Retrospective case enrollment was done at 1 hospital from July 2012 through August 2013. Demographic characteristics, symptom onset, and rotavirus vaccine status were ascertained. Using the self-controlled case-series method, we estimated age-adjusted incidence rate ratios within 1–7, 8–21, and 1–21 days of rotavirus vaccination in children aged 28–275 days at onset of symptoms. In addition, age-matched controls were enrolled for a subset of cases (n = 169), and a secondary analysis was performed.

**Results:**

Three hundred forty-six cases were included in the case-series analysis. Post–dose 1, there were zero intussusception cases within 1–7 days, and 5 cases within 8–21 days of vaccination. Post–dose 2, 15 cases occurred within 1–7 days, and 18 cases within 8–21 days of vaccination. There was no increased risk of intussusception 1–7 days after dose 1 (no cases observed) or dose 2 (relative incidence [RI], 1.71 [95% confidence interval {CI} .83–3.01]). Similarly, there was no increased risk 8–21 days after the first (RI, 4.01 [95% CI, .87–10.56]) or second dose (RI, .96 [95% CI, .52–1.60]). Results were similar for the case-control analysis.

**Conclusions:**

The risk of intussusception in the 21 days after the first or second dose of RV1 was not higher than the background risk among South Africa infants.

**Clinical Trials Registration:**

South African National Clinical Trial Register (DOH-27-0913-4183).

## Teaser

There was no association between intussusception and the first or second dose of rotavirus vaccination among infants in South Africa, and no clustering of cases in any of the risk windows (1‒7, 8‒21, 1‒21 days) after receipt of either dose.

Intussusception, invagination of one part of the intestine into a more distal part, causes bowel obstruction in infants. The majority of cases occur in children <12 months of age, and >90% of cases are idiopathic in nature [1, 2]. The first licensed rotavirus vaccine (Rotashield, Wyeth Lederle Vaccines) was introduced into the routine immunization program in the United States in 1998 and was withdrawn a year later due to an association with an increased risk of intussusception [3]. Large safety and efficacy trials, conducted in high- and middle-income countries, of 2 subsequent oral live-attenuated rotavirus vaccines, Rotarix (RV1; GlaxoSmithKline Biologicals) and RotaTeq (RV5; Merck & Co, Inc), did not show an increased risk of intussusception [4, 5]. In 2009, the World Health Organization recommended inclusion of rotavirus vaccination of infants into all national immunization programs [6].

To date, 98 countries have introduced rotavirus vaccines, and postlicensure studies have demonstrated effectiveness against rotavirus disease under routine use, with substantially decreased hospitalizations and mortality from rotavirus and all-cause acute gastroenteritis [7–9]. Postlicensure studies have also identified a low-level risk of intussusception 1–7 days after the first and/or second dose of rotavirus vaccine administration in some high- and middle-income countries including Mexico, Brazil, Australia, the United Kingdom, and the United States [10–14]. However, there is consensus that the benefits of rotavirus vaccination strongly outweigh this risk [15, 16]. An increased risk of intussusception after administration of RV1 was not seen in 7 lower-income sub-Saharan African countries [17].

The incidence of intussusception varies considerably by region. Thus, it is important to evaluate postlicensure risk following rotavirus vaccination in various settings. South Africa, an upper-middle-income country, was the first African country to introduce rotavirus vaccine into its national immunization program in August 2009. We established hospital-based surveillance for intussusception at 8 hospitals in 6 cities and used the self-controlled case series (SCCS) and case-control methods to evaluate the association between RV1 administration and the risk of intussusception.

## METHODS

### Study Design and Participants

Active prospective surveillance for intussusception was conducted from September 2013 through December 2017 at the Charlotte Maxeke Johannesburg Academic, Chris Hani Baragwanath Academic, East London, Inkosi Albert Luthuli, Red Cross War Memorial Children’s, Steve Biko Academic/Kalafong, Tygerberg, and Universitas hospitals. In addition, we retrospectively enrolled cases diagnosed from July 2012 through August 2013 at Chris Hani Baragwanath Academic Hospital. Children <3 years of age hospitalized with intussusception, defined by Level 1 Brighton Collaboration criteria, were eligible for enrollment [18]. Level 1 of diagnostic certainty requires confirmation by surgical or radiological reduction of the intussusception. In addition, we attempted to enroll 1 nonintussusception hospitalized surgical control for each case, who was age-matched (date of birth ± 90 days), admitted within 90 days of the case, and enrolled at the same hospital. Written informed consent was obtained from a parent or guardian. Approvals were obtained from the ethics committees of the University of the Witwatersrand, University of KwaZulu-Natal, University of Cape Town, University of Stellenbosch, University of the Free State, University of Pretoria, and Walter Sisulu University. The study was registered at the South African National Clinical Trial Register (DOH–27–0913–4183).

### Procedures

For cases, demographic characteristics, clinical signs and symptoms, and confirmation of diagnosis of intussusception were obtained by parent interview and hospital record review. There was no follow-up of cases. For controls, limited information on demographics and diagnosis was abstracted. Vaccination history for cases and controls was ascertained by review of the vaccination card, a copy of which was obtained whenever possible. A case was considered to be human immunodeficiency virus (HIV) exposed if the mother gave a history of being HIV infected or the child’s HIV enzyme-linked immunosorbent assay was reactive; HIV exposure status was not obtained for controls. Cases were considered HIV infected if HIV polymerase chain reaction (PCR) was positive, and HIV-uninfected if HIV PCR was negative or mother’s HIV status was known to be negative.

### Statistical Analysis

In the primary analysis, we used the SCCS method to estimate age-adjusted intussusception incidence rate ratios within 1–7, 8–21, and 1–21 days after each dose of rotavirus vaccination [19]. With this method, the frequency with which intussusception occurred in exposed periods after vaccination is compared to its occurrence in unexposed periods, in cases only. Each case acts as its own control, and no external controls are needed to evaluate risk as the method controls for time-invariant confounding. The pseudo-likelihood method was used to allow for contraindication of rotavirus vaccination after an episode of intussusception; the standard approach assumes that vaccination is not dependent on the occurrence of the event [20]. We limited the analysis to children aged 28–275 days at onset of symptoms, taking into account the minimum and maximum ages at which rotavirus vaccination could have been given. The unexposed period was the period from 28–275 days, excluding the 1–21 days after each vaccination. The onset of intussusception was considered to be the date of onset for the first reported symptom, including onset of refusal to feed, vomiting, or bloody stools. If no onset date was recorded for these symptoms, then date of admission was used. All cases who had their vaccination card reviewed were included in the analysis. If the vaccination card was unavailable, but the parent indicated that the child had not received any vaccines other than those given at birth, the child was included in the analysis and considered not to have received any RV1 doses. Cases without a vaccination card, but who had a history of vaccination, were excluded. Since background incidence of intussusception can vary several-fold during the first 9 months of life, all vaccinated and unvaccinated cases were included in the final analysis to adjust for changes in the background incidence of intussusception by age. Conditional Poisson regression was used to calculate relative incidence by comparing the incidence within the risk window with the incidence in all other observation windows for each case. We controlled for age in the model using 14-day intervals. Confidence intervals (CIs) were derived by bootstrapping with 1000 iterations. For the primary analysis, we calculated that a sample size of 242 case patients of intussusception would be sufficient to detect a relative incidence of >2.5 within 7 days of receiving a dose of rotavirus vaccine, with 80% power at a 5% significance level using the signed root likelihood ratio method [21].

For the secondary case-control analysis, we used a conditional logistic regression model to assess the ratio of the odds that cases were vaccinated within the risk windows to the odds that age-matched controls were vaccinated within those windows. The season of birth and admission as well as geographical variations in the incidence of intussusception and vaccination were adjusted for by matching cases with controls according to date of birth, date of hospitalization, and hospital. A reference date was created for controls, which was the date on which the control was the same age as the case was at the time of symptom onset; thus, each matched case-control set was the same age in the model. Exposure to vaccination with the first or second RV1 dose within the risk windows (1–7, 8–21, and 1–21 days) was determined relative to this reference date. The final model adjusted only for sex, as information on other potential confounders was not collected. *P* values <.05 were considered to be statistically significant. For both the SCCS and case-control analyses, we performed sensitivity analyses using date of admission for intussusception instead of date of symptom onset. All reported *P* values are 2-sided. We used Stata version 12.1 software for all analyses.

## RESULTS

**Figure 1.**
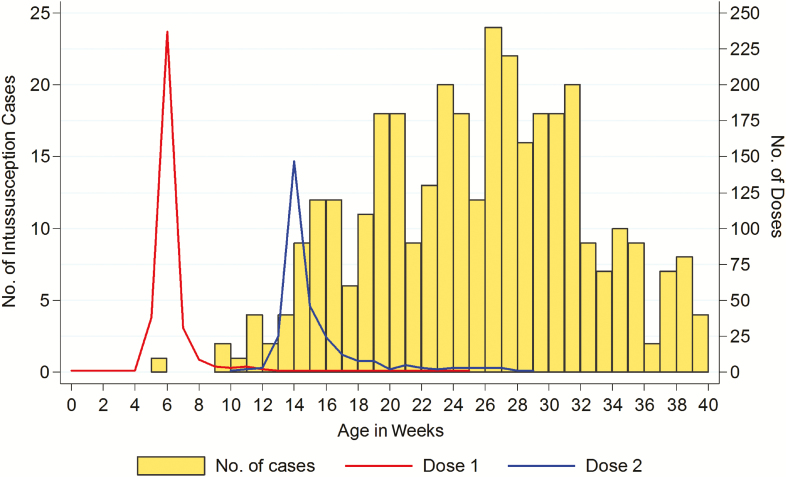
Age at rotavirus (Rotarix [RV1]) vaccination and at onset of intussusception symptoms among cases.

A total of 474 intussusception cases <3 years of age were enrolled across the 8 sites during the study period, of whom 374 (79%) were aged 28–275 days at onset of symptoms. Of these, 346 (93%) had a vaccination status available and were included in the primary analysis (Table 1). There were no significant differences in age at symptom onset, sex, or race between children with and without an available vaccination history (Supplementary Appendix). The median age of included cases was 26 weeks (interquartile range [IQR], 20–30 weeks), 180 (52%) were male, and 282 (82%) were of black race. HIV exposure status was available for 184 cases (53%), of whom 32 (17%) were born to HIV-infected mothers. HIV infection was confirmed in only 1 case. The median time between onset of symptoms and hospitalization was 3 days (IQR, 1–4 days). Vaccine coverage was high; only 10 (3%) cases were unvaccinated, 40 (12%) had received 1 dose, and 296 (85%) had received 2 doses of RV1. The first and second doses of RV1 were given in a timely manner in the majority of the cases, at a median age of 6 weeks (IQR, 6–7 [range, 0–26 weeks]) and 15 weeks (IQR, 14–16 [range, 11–29 weeks]) of age, respectively (Figure 1). Six patients received a dose of RV1 after their intussusception, despite this being contraindicated.

**Table 1.** Enrollment Periods and Number of Intussusception Cases Included in the Self-controlled Case-series Analysis, According to Hospital

Hospital	City, Province	Enrollment Period	Cases Aged <3 y^a^ (n = 474)	Cases Aged 28–275 d^a^ (n = 374)	Cases Aged 28–275 d^a^ With Vaccination Status Available (n = 346)
Chris Hani Baragwanath Academic Hospital	Johannesburg, Gauteng	Jul 2012–Dec 2017^b^	119 (25)	93 (25)	83 (24)
Charlotte Maxeke Johannesburg Academic Hospital	Johannesburg, Gauteng	Feb 2014–Dec 2017	42 (9)	31 (8)	26 (8)
Steve Biko Academic Hospital/ Kalafong Hospital	Pretoria, Gauteng	Oct 2013–Dec 2017	92 (19)	74 (20)	74 (21)
Universitas Hospital	Bloemfontein, Free State	Oct 2014–Dec 2017	23 (5)	22 (6)	22 (6)
Inkosi Albert Luthuli Hospital	Durban, KwaZulu-Natal	Dec 2014–Dec 2017	60 (13)	48 (13)	36 (10)
East London Hospital Complex	East London, Eastern Cape	Sept 2013–Dec 2017	29 (6)	26 (7)	26 (8)
Tygerberg Hospital	Cape Town, Western Cape	Apr 2014–Dec 2017	53 (11)	39 (10)	38 (11)
Red Cross Children’s Hospital	Cape Town, Western Cape	Apr 2014–Dec 2017	56 (12)	41 (11)	41 (12)

Data are presented as no. (%).

^a^Age is calculated according to onset of symptoms, including refusal to feed, vomiting, or bloody stools.

^b^Eighteen intussusception cases were enrolled retrospectively from July 2012 through August 2013. Prospective enrollment commenced September 2013.

### Case-series Analysis

**Figure 2.**
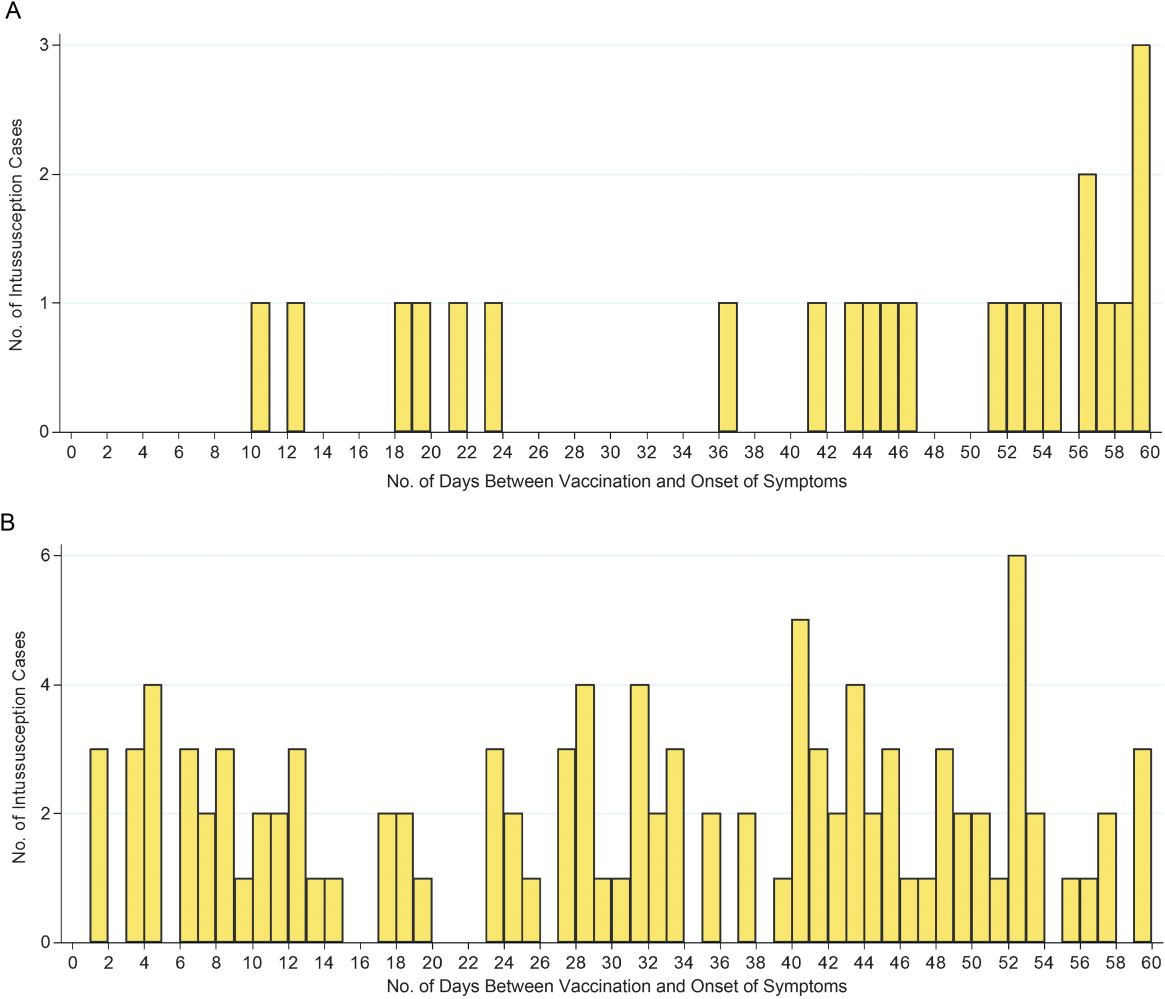
Cases of intussusception occurring during the first 60 days after dose 1 (*A*) and dose 2 (*B*) of rotavirus vaccine (Rotarix [RV1]). Three hundred thirteen intussusception cases occurred >60 days after dose 1; 183 cases occurred >60 days after dose 2.

There were zero intussusception cases within 1–7 days, and 5 cases within 8–21 days of vaccination with the first RV1 dose (Figure 2A). After vaccination with the second RV1 dose, 15 cases occurred within 1–7 days, and 18 cases within 8–21 days of vaccination (Figure 2B). Compared to the background risk, there was no increased risk of intussusception 1–7 days after dose 1 (no cases observed) or dose 2 (relative incidence [RI], 1.71 [95% CI, .83–3.01]). Similarly, there was no increased risk 8–21 days after the first dose (RI, 4.01 [95% CI, .87–10.56]) or second dose (RI, .96 [95% CI, .52–1.60]; Table 2). RI estimates for all risk windows were similar when using date of admission for intussusception, instead of date of symptom onset (Supplementary Appendix). The HIV-infected case had intussusception symptom onset 21 days after the first dose of RV1 and did not receive a second RV1 dose.

**Table 2.** Relative Incidence of Intussusception^a^ in the Risk Windows After the First and Second Doses of Monovalent Rotavirus Vaccine: Self-controlled Case-series Analysis

Dose of RV1	Risk Period	No. of Cases^b^	Relative Incidence^c^ (95% CI)
1	Days 1–7	0	0
	Days 8–21	5	4.01 (.87–10.56)
	Days 1–21	5	3.14 (.66–8.49)
2	Days 1–7	15	1.71 (.83–3.01)
	Days 8–21	18	.96 (.52–1.60)
	Days 1–21	33	1.19 (.74–1.85)

Abbreviations: CI, confidence interval; RV1, Rotarix.

^a^Onset of intussusception was considered to be the date of onset for the first reported symptom, including onset of refusal to feed, vomiting, or bloody stools.

^b^Eight to 21 days post–dose 1: human immunodeficiency virus (HIV) infected, n = 1; HIV-exposed but uninfected (HEU), n = 2; missing HIV status, n = 2. One to 7 days post–dose 2: HEU, n = 4; HIV unexposed, n = 4; missing HIV status, n = 7. Eight to 21 days post–dose 2: HIV unexposed, n = 5; missing HIV status, n = 13.

^c^Relative incidence is a ratio of the incidence within the risk window vs the incidence in all other observation windows for each infant, calculated with the use of conditional Poisson regression.

### Case-control Analysis

For the secondary analysis, 169 intussusception cases had a correctly matched control and were included in the case-control evaluation. Eighty-five (50%) cases vs 126 (75%) controls were male (*P* < .001), and 141 (83%) cases vs 139 (82%) controls were of black race (*P* = .992). Two (1%) controls were unvaccinated, 33 (20%) had received 1 dose of RV1, and 134 (79%) had received 2 doses of RV1. The most common diagnoses among controls were inguinal hernia (33%), anorectal malformation (22%), Hirschsprung disease (5%), and umbilical hernia (4%); each of these conditions had a higher prevalence in males. The odds of intussusception in the first 21 days after the first or second dose of RV1 were not significantly different in cases and controls (odds ratio [OR], 2.00 [95% CI, .18–22.06] vs 1.73 [95% CI, .60–5.01], respectively; Table 3); nor in any of the other the risk windows. ORs were similar for all risk windows when using date of admission rather than date of symptom onset (Supplementary Appendix). There were no significant differences in sex, race, age at symptom onset, age at vaccination, and vaccine coverage between cases who had matched controls and were included in the case-control analysis, and those who were not included in this analysis (data not shown).

**Table 3.** Odds of Intussusception^a^ in the Risk Windows After the First and Second Doses of Monovalent Rotavirus Vaccine: Case-control Analysis

Dose of RV1	Risk Period	No. of Cases in Risk Window^b^ (n = 169)	No. of Controls in Risk Window^b^ (n = 169)	Odds Ratio (95% CI)
1	Days 1–7	0	0	…
	Days 8–21	2	1	2.00 (.18–22.06)
	Days 1–21	2	1	2.00 (.18–22.06)
2	Days 1–7	6	3	2.22 (.50–9.78)
	Days 8–21	10	10	1.51 (.40–5.63)
	Days 1–21	16	13	1.92 (.68–5.40)

Abbreviations: CI, confidence interval; RV1, Rotarix.

^a^Onset of intussusception was considered to be the date of onset for the first reported symptom, including onset of refusal to feed, vomiting, or bloody stools.

^b^Risk window is number of days prior to the reference date (date of symptom onset in cases; date on which controls were the same age as cases were at symptom onset).

## DISCUSSION

We did not find a significant association between intussusception and the first or second dose of RV1 vaccination among infants in South Africa, and no clustering of cases occurred in any of the risk windows (1‒7 days, 8‒21 days, or 1‒21 days) after receipt of either dose. This is the first study assessing the risk of intussusception after rotavirus vaccine in an upper-middle-income African country and using a schedule in which the first dose is given at 6 weeks and the second dose at 14 weeks of age, rather than 10 weeks as in other African countries. Our results are in keeping with a recent evaluation of intussusception after RV1 vaccination in low-income and low-middle-income countries in sub-Saharan Africa, where the risk of intussusception in association with either the first or second dose of RV1 was not higher than the background risk [22]. However, our findings contrast to those in Brazil and Mexico, 2 other upper-middle-income countries, where an increase in risk of intussusception was observed in the first 7 days after the first dose of RV1 in Mexico (RI, 5.3; OR, 5.8) and in the first 7 days after the second dose in Brazil (RI, 2.6; OR, 1.9) [10]. Studies from high-income countries including the United States, Australia, and the United Kingdom have also shown an association between intussusception and rotavirus vaccination, mainly in the first 7 days after the first dose of RV1 (ranging from 6.8 to 13.8) with a smaller risk after the second dose [11, 13, 14].

The RV1 schedule in South Africa is more in line with the rest of Africa and other developing countries, where vaccination is recommended at 6 and 10 weeks of age, rather than at 2 and 4 months of age in high- and middle-income countries where intussusception risk has been documented. Intussusception is relatively uncommon at the young age at which the first dose of RV1 is administered in African countries and even at 14 weeks of age when the second dose is given in South Africa. In our study, only 1 case of intussusception occurred at or before 6 weeks of age, and 23 cases at or before 14 weeks of age. Thus, the lack of an association between rotavirus vaccination and intussusception may be due to administration at an age when the risk of natural intussusception is low. There are also differences in the protection afforded by the vaccine, with RV1 vaccine efficacy against severe rotavirus disease shown to be higher in high- and middle-income Latin American countries compared with that in South Africa [5, 23]. Immune responses to rotavirus vaccination and fecal shedding of vaccine-virus strains after the first rotavirus vaccine dose in lower-income countries are also generally lower than they are in high-income countries [24]. Thus, the lack of intussusception risk postvaccination may be explained by reduced intestinal replication of the vaccine virus in lower-income countries, although the mechanism of vaccine-induced intussusception and potential correlation with immune response has not been elucidated. RV1 efficacy in South Africa was higher than that observed in Malawi (77% vs 49%, respectively) during the phase 3 study [23], but our study shows that whatever the reason for this higher efficacy, such as increased intestinal replication, it has not produced a significant risk of intussusception.

Other factors may also play a part in the differential risk of intussusception between low- and middle-countries in Africa and higher-income countries. Coadministration with oral poliovirus vaccine (OPV) has been shown to decrease the immunogenicity of the first dose of RV1 and, potentially, the replication of the vaccine virus [25]. In South Africa, children receive OPV concurrent with RV1 at the 6-week immunization visit, and inactivated poliovirus vaccine at both the 6- and 14-week visits. Differences in levels of antirotavirus maternal antibodies transferred to the infant via the placenta and in breast milk, the infant gut microbiome, nutrition, and host genetics may affect immune responses to the rotavirus vaccine and may thus also potentially have an impact on the risk of intussusception [24].

South Africa has a high HIV prevalence among women of child-bearing age, with just over 30% of infants born to HIV-infected mothers [26]. There is some evidence suggesting that antibody responses to vaccines—for example, pertussis and pneumococcus—may be more robust in HIV-exposed but uninfected (HEU) infants, possibly due to lower maternally derived antibody levels and less inhibition of infant responses [27]. In a South African effectiveness study, the adjusted vaccine effectiveness point estimate after the first dose of RV1 was higher in HEU than in HIV-unexposed children, suggesting a more robust immune response in these infants, possibly due to lower transplacental transmission of rotavirus-specific antibodies in HEU children [28]. If postvaccination intussusception risk is linked to intestinal replication of the vaccine virus, then HIV-exposed infants may theoretically be at increased risk after the first dose. Although we were not powered to compare intussusception risk by HIV exposure status, we did not observe any cases of intussusception in the first 7 days after the first dose, providing some reassurance that this hypothesis of increased risk in HIV-exposed infants does not seem to hold true.

Our study had some limitations. Parents often have to travel considerable distances to bring their child to the hospital, especially in the poorer provinces of South Africa, and it is possible that we may have missed cases of intussusception due to infants dying before reaching the hospital. However, we have no reason to believe that this would not be randomly distributed in relation to timing of vaccination, and thus was unlikely to cause bias. We could not obtain vaccination status on all patients, but this was available for >90% of cases, and there were no significant differences for key parameters such as age between cases with and without vaccination status. Cases enrolled retrospectively could potentially introduce bias; however, information was reliably obtained through review of the hospital record and the vaccination status was obtained from the parent. All controls were enrolled prospectively. Despite not reaching statistical significance, the point estimate for risk of intussusception in the 8‒21 days after the first dose was elevated. It is possible that the nonsignificance was merely due to the sample size; however, biologically it would be difficult to explain an increase in this risk period without an increased risk in the first 7 days. For the case-control analysis, we did not manage to enroll a control for all intussusception cases, and less than half of cases had an appropriately matched control, limiting our sample size. In addition, we collected limited data on the controls, and thus could only adjust for sex in the analysis and not all potential confounders. Nevertheless, these results were similar to those of the SCCS analysis, albeit with wider CIs. We tried to include cases from as many of the major referral hospitals in South Africa as possible. Although only 5 of the 9 provinces were represented, the recruiting hospitals drew cases from areas with diverse socioeconomic characteristics, and we believe that the results are generalizable to other areas of South Africa.

In conclusion, the risk of intussusception in the 1–7 and 8–21 days after the first or second dose of the monovalent oral rotavirus vaccine was not found to be higher than the background risk of intussusception among infants in South Africa. Postlicensure impact studies in our country have shown significant decreases in rotavirus-specific hospitalizations, with a 61%–69% reduction in the first 2 years after vaccine introduction, and sustained reductions in all-cause diarrheal hospitalizations in children <2 years of age [29, 30]. The absence of an increased risk of intussusception after rotavirus vaccination in an upper-middle-income African country is reassuring.

## Supplementary Data

Supplementary materials are available at *Clinical Infectious Diseases* online. Consisting of data provided by the authors to benefit the reader, the posted materials are not copyedited and are the sole responsibility of the authors, so questions or comments should be addressed to the corresponding author.

ciz431_suppl_Supplementary_Appendix

## Notes

***Author contributions.*** Conception and design of the study: M. J. G., S. A. M., U. D. P. Acquisition of data: M. A., M. C., S. C., C. d. V., M. K., S. M. G., J. L., S. M., A. M. Analysis of data: M. J. G., J. E. T., N. A. Interpretation of data: M. J. G., J. E. T., N. A., S. A. M., U. D. P. Drafting of article: M. J. G. Critically revising drafts of article and final approval of submitted version: all authors.

***Acknowledgments.*** The authors thank all of the clinical and administrative staff members in the Paediatric Surgery Departments who assisted with the study at the recruiting hospitals; their study coordinator Anna Welthagen (Department of Paediatric Surgery, University of the Witwatersrand); and Manish Patel (Centers for Disease Control and Prevention [CDC]), who assisted with the initial funding proposal.

***Disclaimer.*** The content of this work is solely the responsibility of the authors and does not necessarily represent the official views of the National Institutes of Health (NIH) or the CDC. The funding source had no role in the writing of the manuscript or the decision to submit it for publication. The authors have not been paid to write this article by a pharmaceutical company or other agency. The corresponding author had full access to all the data in the study and had final responsibility for the decision to submit for publication.

***Financial support.*** Support for this project was provided by the Bill & Melinda Gates Foundation (BMGF) (grant number OPP1066762). M. J. G. received support from an Emerging Global Leader Award from the Fogarty International Center of the NIH (grant number K43TW010382).

***Potential conflicts of interest.*** M. J. G. has received grants from BMGF, NIH, and PATH. S. A. M. has received grants from BMGF, Pfizer, GlaxoSmithKline, and Minervax, and personal fees from BMGF. All other authors report no potential conflicts of interest. All authors have submitted the ICMJE Form for Disclosure of Potential Conflicts of Interest. Conflicts that the editors consider relevant to the content of the manuscript have been disclosed.

Presented in part: 13th International Rotavirus Symposium, Minsk, Belarus, 29‒31 August 2018.
