# Supplementary material for: Evaluation of Intussusception After Oral Monovalent Rotavirus Vaccination in South Africa
Source: Clin Infect Dis. 2019 May 24;70(8):1606–12. doi: 10.1093/cid/ciz431 (PMC7146001; doi:10.1093/cid/ciz431)
Supplement: ciz431_suppl_Supplementary_Appendix [file ciz431_suppl_supplementary_appendix.docx]

**Appendix**

**Supplementary Table 1: Characteristics of intussusception cases with (included in analysis) and without (excluded from analysis) vaccination status available**

| Characteristic | With vaccination status available  N=346 | Without vaccination status available  N=28 | p-value |
| --- | --- | --- | --- |
| Age in weeks (median, IQR) | 26 (20‒30) | 26 (23‒31) | 0.540^*^ |
| Male (n, %) | 180 (52) | 16 (57) | 0.602^#^ |
| Black race (n, %) | 282 (82) | 26 (93) | 0.195^ |
| HIV-infected (n/N, %) | 1/184 (0.5) | 0/16 (0) | ‒ |
| HIV-exposed (n/N, %) | 32/184 (17) | 5/16 (31) | 0.183^ |
| Time between symptom onset and hospitalization (median, IQR) | 3 (1‒4) | 3 (1‒4) | 0.892^*^ |

IQR – interquartile range;

^*^Wilcoxon rank-sum

^#^Chi-squared

^Fisher’s exact

**Supplementary Table 2: Relative incidence of intussusception^#^ in the risk windows after the first and second doses of monovalent rotavirus vaccine – self-controlled case series analysis**

| Dose of RV1 | Risk period | No of cases | Relative incidence (95% CI) |
| --- | --- | --- | --- |
| 1 | Day 1–7 | 0 | 0 |
|  | Day 8–21 | 4 | 3·23 (0·45–11·92) |
|  | Day 1–21 | 4 | 2·50 (0·31–7·89) |
| 2 | Day 1–7 | 9 | 0·98 (0·38–1·94) |
|  | Day 8–21 | 22 | 1·13 (0·60–1·89) |
|  | Day 1–21 | 31 | 1·08 (0·65–1·71) |

Relative incidence is a ratio of the incidence within the risk window versus the incidence in all other observation windows for each infant, calculated with the use of conditional Poisson regression.

**^#^**The onset of intussusception in the case was considered to be the date of admission.

**Supplementary Table 3: Odds of intussusception^#^ in the risk windows after the first and second doses of monovalent rotavirus vaccine – case-control analysis**

| Dose of RV1 | Risk period | No of cases in risk window*  N=169 | No of controls in risk window*  N=169 | Odds ratio (95% CI) |
| --- | --- | --- | --- | --- |
| 1 | Day 1–7 | 0 | 0 | ­­– |
|  | Day 8–21 | 2 | 1 | 2·00 (0·18–22·06) |
|  | Day 1–21 | 2 | 1 | 2·00 (0·18–22·06) |
| 2 | Day 1–7 | 3 | 1 | 4·57 (0·42–49·64) |
|  | Day 8–21 | 12 | 12 | 1·29 (0·39–5·63) |
|  | Day 1–21 | 15 | 13 | 1·73 (0·60–5·01) |

*Risk window is number of days prior to the reference date (date of admission in cases; date on which controls were the same age as cases were on admission)

**^#^**The onset of intussusception in the case was considered to be the date of admission.
